# Kimura disease, a rare cause of inguinal lymphadenopathy: A case report

**DOI:** 10.3389/fmed.2022.1023804

**Published:** 2022-09-23

**Authors:** Xianwen Hu, Xue Li, Changwei Yang, Dandan Li, Jiong Cai, Pan Wang

**Affiliations:** ^1^Department of Nuclear Medicine, Affiliated Hospital of Zunyi Medical University, Zunyi, China; ^2^Department of Obstetrics, Zunyi Hospital of Traditional Chinese Medicine, Zunyi, China

**Keywords:** Kimura’s disease, inguinal lymphadenopathy, PET/CT, magnetic resonance imaging, flfluoro18-labeled deoxyglucose

## Abstract

Kimura’s disease (KD) is a rare chronic granulomatous disease of unknown etiology that mainly involves damage to lymph nodes, soft tissues, and salivary glands. The clinical symptoms are mainly painless subcutaneous soft tissue masses, often involving head and neck lymph nodes and salivary glands, and are mainly characterized by diffuse eosinophilic infiltration, lymphocyte, and vascular proliferation. There are few reports in the literature that KD affects only inguinal lymph nodes. We report in this study a 41-year-old male patient who presented to the hospital for medical help with soft tissue masses in the groin. Magnetic resonance imaging (MRI) showed multiple abnormal soft tissue nodules around the iliac vessels in the left groin, and a contrast-enhanced scan showed obvious homogeneous enhancement. Diffusion-weighted imaging showed limited movement of water molecules and showed an obvious high signal. Fluoro18-labeled deoxyglucose positron emission tomography/computed tomography (^18^F-FDG PET/CT) was recommended for further evaluation of the patient’s general condition, and the results showed that except for the radioactive uptake in the lesions in the left groin region, no obvious abnormality was found in the rest of the body. Based on these imaging findings, the patient was first suspected to have malignant lesions, and then the patient underwent histopathological examination, which was confirmed to be KD. Our case study suggests that KD affects only the inguinal lymph nodes is rare and should be considered as one of the imaging differential diagnoses for lymphadenopathy such as lymphoma, metastases, and Castleman’s disease.

## Case description

**FIGURE 1:**
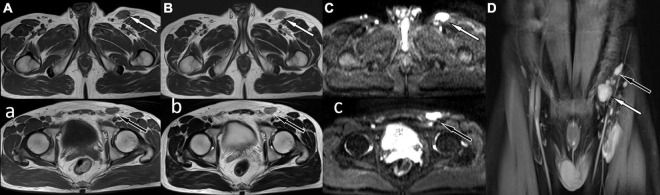
Magnetic resonance imaging (MRI) showed abnormal soft tissue nodules around the iliac vessels in the left groin, T1WI sequence was isointense with muscle tissue **(A,a)** white arrow levels the plane of the greater trochanter, black arrow levels the plane of the femoral head), and T2WI was slightly hyperintense **(B,b)**. Diffusion-weighted imaging showed obvious high signal **(C,c)**, and contrast-enhanced scan showed obvious homogeneous enhancement **(D)**.

**FIGURE 2:**
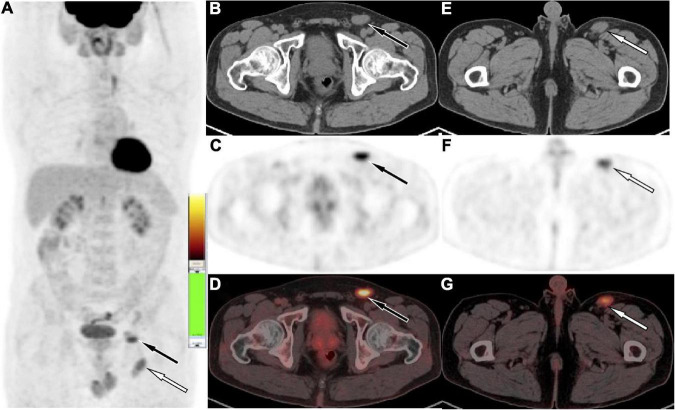
Fluoro18-labeled deoxyglucose positron emission tomography/computed tomography (^18^F-FDG PET/CT) of the patient, the maximum intensity projection [MIP, **(A)**] showed multiple mild to moderate radioactive uptake foci in the left inguinal region (white arrow levels the plane of the greater trochanter, black arrow levels the plane of the femoral head). The axial figures in the left inguinal region at the femoral head level [**(B)** CT; **(C)** PET; **(D)** PET/CT fusion] and greater trochanter level [**(E)** CT; **(F)** PET; **(G)** PET/CT fusion] showed soft tissue nodules with radioactive uptake, with SUVmax of 5.3 (black arrow) and 4.5 (white arrow), respectively.

**FIGURE 3:**
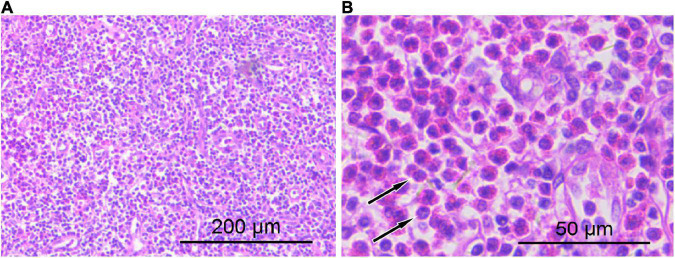
Hematoxylin–eosin staining showed lymphocyte proliferation in the lesions with diffuse eosinophil infiltration [**(A)** 100 × magnification; **(B)** 400 × magnification arrows indicate eosinophilic microabscesses].

A 41-year-old man came to our hospital for medical help because of the discovery of groin masses for over 1 month. Physical examination revealed that two soft tissue nodules with a length of about 1.0 cm were palpable in the left inguinal region, which was tough in quality, with good mobility and no obvious tenderness. No obvious positive signs were seen in other parts of the body. The blood routine results revealed that the eosinophil count was increased, with a value of 0.78 × 10^9^/L, and the rest leukocyte and platelet counts were all within the normal reference value range. His immunoglobulin (Ig) E level was slightly elevated, with a value of 204 IU/ml (the reference value was less than 165 IU/ml), and IgG, IgM, and IgA were all within the normal reference range. Magnetic resonance imaging (MRI) was recommended for the nature of these soft tissue masses, which showed isointensity with muscle tissue on T1WI and T2WI (as shown in Figure 1). Diffusion-weighted imaging (DWI) showed hyperintensity, and a contrast-enhanced scan showed obvious homogeneous enhancement, which was considered as an enlarged lymph node, but the benign and malignant lesions could not be determined. To further systematically evaluate the patient’s whole-body condition, the patient underwent ^18^F-FDG PET/CT examination (as shown in Figure 2), of which results showed that there were multiple nodules with increased radioactivity uptake of different sizes in the left inguinal region, with maximum standard uptake value (SUVmax) ranging from 4.5 to 5.3, and no obvious abnormal radioactive uptake foci were found in the rest of the body. The lesion was limited and surgical resection was feasible for this patient, so he underwent left groin mass excision under local anesthesia. Postoperative pathological and immunohistochemical examination (as shown in Figure 3) of the excised mass showed that it was an eosinophilic lymphogranuloma, that is, Kimura’s disease (KD), and the gene test result was wild type. Based on the established KD diagnosis, the patient received additional oral prednisone for 1 year postoperatively. The patient was followed up by regular telephone calls for 3 years, and there was no sign of recurrence.

## Discussion

Kimura disease (KD), also known as eosinophilic lymphogranulomatosis, is a rare benign chronic lymphoproliferative disorder originating in the dermis, subcutaneous tissue, and lymph nodes, which is more common in Asian adult men (1, 2). KD often occurs in the head and neck lymph nodes, easily involving the salivary glands, and rarely occurs in the inguinal lymph nodes (3, 4). At present, the etiology of KD is not clear, which may be related to allergic reactions caused by candida albicans, parasites or viruses, arthropod bite, endocrine disorders, autoimmune diseases that change the regulatory T-cell immune response, or induce IgE-induced type I hypersensitivity, resulting in the release of eosinophils such as interleukin-4 and interleukin-5, which eventually leads to the deposition of eosinophils in the diseased tissue (5, 6). The disease lacks characteristic clinical manifestations and usually presents as single or multiple painless masses in the affected area, a few may be accompanied by skin itching, and unilateral disease is more common (7). The patient we present is a middle-aged male with unilateral painless lymphadenopathy with elevated eosinophils on laboratory tests, consistent with features of KD, but our patient presented with only inguinal lymph node involvement, which is rare.

Imaging examinations including CT, MRI, and PET/CT play an important role in the localization and characterization of KD. On CT, the swollen lymph nodes involved are more uniform in density, and there are few low-density cystic necrosis areas and high-density calcifications (3). One study has divided KD into two types according to the CT findings of the lesions, namely nodular type with clear edge and uniform density and diffuse swelling type with unclear edge and infiltration of surrounding subcutaneous fat (8). The main manifestations of nodular type are homogeneous isomuscular signal on T1WI, slightly high-to-high signal on T2WI and DWI. Diffuse swelling type is mainly manifested as inhomogeneous hypointense on T1WI with a relatively blurred boundary of the lesion, and uneven iso-to-high signal on T2WI, and contrast-enhanced scans show different degrees of enhancement (4). Our patient presented as soft tissue masses with well-defined borders and uniform density/signal, no cystic necrosis or calcification, and contrast-enhanced scan showed obvious uniform enhancement, consistent with nodular type KD. There are currently few PET/CT studies on KD, and a previous study (9) showed a SUVmax of 4.0–4.5 for KD lesions, which are consistent with our patient (SUVmax of 4.5–5.3).

The location of the lymph nodes in the groin area is superficial, and lymphadenopathy is one of the common clinical signs, and the etiology includes infectious and tumorous lesions. KD involving inguinal lymph nodes mainly needs to be differentiated from lymphoma, lymph node tuberculosis, metastases, and giant lymph node hyperplasia (Castleman disease, CD). Lymphoma has a short course of disease, with fever, weight loss in a short period of time, and other cachexia, most of which are bilateral and multiregional. The lymph nodes in the affected area often tend to fuse and show mild to moderate homogeneous enhancement after contrast enhancement (3, 10). ^18^F-FDG PET/CT has been widely used in the diagnosis, staging, and evaluation of the degree of malignancy of lymphoma. According to the pathological nature of lymphoma, it can show different degrees of radioactive uptake. Based on our case and previous reports, the radioactive uptake of KD is similar to that of indolent lymphoma, making it difficult to differentiate (9, 11). Lymph node tuberculosis often has clinical manifestations of low fever, fatigue, night sweats, and increased erythrocyte sedimentation rate, which is more common in young people, and more secondary or associated with tuberculosis. MRI showed uneven signals in the long T1 and T2 of the lesion lymph nodes, and contrast-enhanced scan showed thick-walled annular enhancement, easy adhesion and fusion, and a “multilocular sign” with relative specificity (12). On ^18^F-FDG PET/CT images, lymphatic tuberculosis often showed lymphadenopathy with heterogeneous density, low-density necrosis, and/or high-density calcification foci. Active lymphatic tuberculosis often showed high radiation uptake, and the central necrotic area showed radiation distribution defect (13). Inguinal lymph node metastasis is one of the common metastatic routes of abdominal and pelvic malignant tumors. It is characterized by enlarged lymph nodes, uneven density or signal, which can be fused into masses, and lymph nodes with cystic necrosis show thin-walled heterogeneous enhancement on contrast-enhanced scans (14). Lymph node metastases have similar biological characteristics to primary tumors and the metabolic activity of most malignant tumors is significantly increased, so lymph node metastases show high uptake of ^18^ F-FDG. Furthermore, ^18^F-FDG PET/CT imaging has high sensitivity and specificity for the diagnosis of lymph node metastases, especially for small lymph nodes with no obvious changes in size, shape, and density (15). Castleman disease can occur in any site with lymph nodes, and it is rare in the groin. MRI plain scan showed that the lesions were isointense on T1WI, hyperintensity on T2WI and DWI, relatively uniform in texture, rare in necrosis, and uniform on T1WI-enhanced signal. In some cases, obviously thickened and tortuous feeding vessels with flow void signal were seen in or around the lesions, and radial or fissure-like unenhanced areas may also occasionally be seen within the lesion, which may be thickened hyalinized collagen fibers (16). The metabolism of CD in ^18^F-FDG PET/CT was slightly increased, and delayed imaging showed no significant changes. If the SUVmax of the diseased lymph nodes were increased, which indicated that the lesion was evolving and might develop into lymphoma (17). Moreover, angiolymphoid hyperplasia with eosinophilia (ALHE), hemangioma, etc., are also one of the rare causes of inguinal lymphadenopathy. ALHE is more common in young women, often presenting as a single subcutaneous mass with clear edges, erythematous, brittle skin, easy to bleed, eosinophils, and elevated serum IgE are rare, and most of them are not accompanied by lymphadenopathy, which can be differentiated from KD (18). Hemangiomas are often seen with round phleboliths, which are not difficult to differentiate. Our patient presented with multiple enlarged lymph nodes confined to the left groin with homogeneous density or signal, marked homogeneous enhancement on contrast-enhanced scan, moderate uptake of ^18^F-FDG, which partially overlapped with the imaging findings of CD and indolent lymphoma, combined with laboratory findings of elevated eosinophil counts, making the diagnosis of KD possible.

The diagnosis of KD still requires histopathological examination, including extensive lymphoid follicle-like structures are seen in the lesions, and a large number of eosinophils infiltrate the lymphoid follicles and the formation of eosinophilic microabscesses are the characteristic manifestations of the diagnosis of this disease (7). The treatment of KD currently mainly includes surgical resection, glucocorticoid therapy, radiotherapy, and chemotherapy, which is sensitive to glucocorticoid therapy and chemotherapy, but it is prone to recurrence after drug withdrawal. A previous study revealed that the combination of surgical resection and postoperative radiotherapy can reduce the local recurrence rate of KD (19). Our patient had a good prognosis after combined glucocorticoid therapy after surgical resection of the lesion, and no disease recurrence has been found in the 3-year follow-up.

## Conclusion

Kimura’s disease affects only the inguinal lymph nodes is rare and should be considered as one of the imaging differential diagnoses for lymphadenopathy such as lymphoma, metastases, and Castleman’s disease. Combined glucocorticoid therapy after surgical resection of the lesion can reduce local recurrence in patients with KD.

## Data availability statement

The original contributions presented in this study are included in the article/supplementary material, further inquiries can be directed to the corresponding authors.

## Ethics statement

Written informed consent was obtained from the individual(s) for the publication of any potentially identifiable images or data included in this article.

## Author contributions

JC and PW: funding acquisition. DL: investigation. XL and CY: methodology. XH: writing-original draft. XH, PW, and JC: writing-review and editing. All authors contributed to the article and approved the submitted version.
